# Supplementary material for: Effect of Water Hardness on Catechin and Caffeine Content in Green Tea Infusions
Source: Molecules. 2021 Jun 8;26(12):3485. doi: 10.3390/molecules26123485 (PMC8229914; doi:10.3390/molecules26123485)
Supplement: Supplementary file 1 [file molecules-26-03485-s001.zip › molecules-1215001-supplementary.pdf]

Supplemental Materials:

Effect of water hardness on catechin and caffeine levels in green tea infusions

Mica Cabrera, Faizah Taher, Alendre Llantada, Quyen Do, Tyeshia Sapp, Monika Sommerhalter*

Department of Chemistry and Biochemistry, California State University East Bay, Hayward CA 94542, United States

HPLC Method Development:

Importance of acidic modifier to reduce peak tailing in pentafluorophenyl-based separations of green tea catechins

Most studies on the quantification of food flavonoids including green tea catechins employ reverse phase C18 columns. Since catechins have aromatic ring components, the use of a column with pentafluorophenyl groups could be a useful alternative. Typical chromatograms obtained with these two different stationary phases are shown in Figure S1. For a direct comparison, the same instrument settings were applied. An Agilent Technologies 1260 Infinity instrument was used. Both columns have the same dimension (250 x 4.6 mm) and particle size (4 µm). The detection wavelength of the diode array detector was 280 nm and the injection volume was 2 µL. The temperature was controlled at 25°C. Solvent A was 0.1 % formic acid in HPLC-grade water and solvent B was acetonitrile with 0.1 % formic acid. The following gradient was used: 0-5 min (10% B), 5-50 min (10% to 25 % B), 51-59 min (80 % B), 60-90 min (10% B). The flow rate was set to 0.5 mL/min.

Both column materials can be used to detect and quantify caffeine as well as the major green tea catechins EGCG, EGC, EC and ECG. Caffeine has a very similar elution time on both

column materials (18.65 ± 0.03 min on the C18 column and 18.20 ± 0.04 min on the PFP column), but the PFP stationary phase resulted in longer retention times for all catechins resulting in EGC eluting after caffeine (Fig. 1C). The use of the PFP column resulted in larger peak separations, but the peak widths are larger compared to the C18 column. The non-epicatechins GCG and CG were most affected by line broadening and peak tailing. Details on retention times, peak widths, peak symmetries, and calibration curve parameters are summarized in Supplemental Tables S1 and S2.

Without adjusting the mobile phase, the PFP column was inferior to the C18 column. An increase in the acidic modifier formic acid from 0.1% to 2%, however, improved peak symmetry and decreased peak width. The beneficial effect of increasing formic acid content in the mobile phase was also seen in working with the shorter InfinityLab Poroshell PFP column (2.1 x 50 mm, 1.9 µm particle size). Figure S2 shows example chromatograms of a standard mixture obtained with the following gradient: 0 - 1 min (5% B), 1- 23 min (5-18% B linear gradient), 24- 28 min (90% B), 29-40 min (5% B). Solvent A was HPLC-grade water with formic acid and solvent B was acetonitrile with formic acid. The concentration of formic acid was varied from 0.1% to 2% v/v. The injection volume was 1 µL, the flow rate 0.5 mL/min and the temperature of the column was controlled at 25 °C. The detector was set to 280 nm.

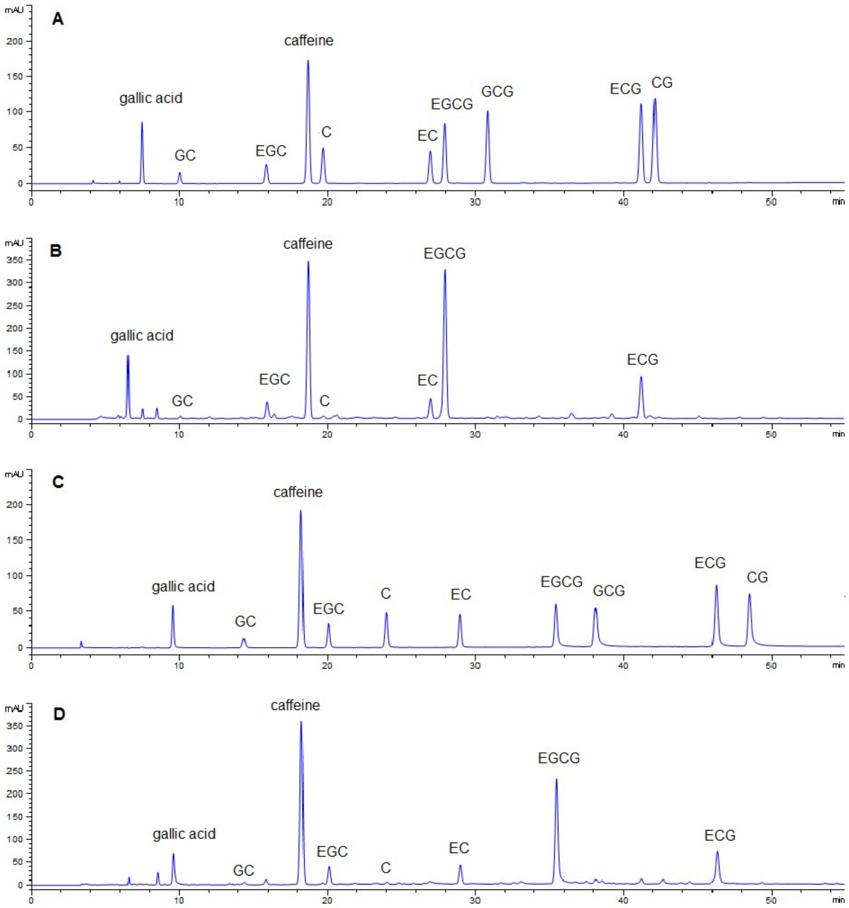

Figure S1: HPLC chromatograms recorded with C18 and PFP columns. An InfinityLab Poroshell 120 EC-18 column (4.6 x 250 mm, particle size 4 µm) (A and B) and an InfinityLab Poroshell PFP column (4.6 x 250, particle size 4 µm) (C and D) were used. A standard mixture (A and C) and a Lipton green tea prepared with deionized water (B and D) were injected.

Supplemental Table S1: HPLC parameters for the C18 Poroshell 120 column (4.6 x 250 mm, 4 µm particle size)

Compound	Retention time (min)	Peak width*	Peak symmetry	Resolution	Slope**	Intercept	R-square value	LOD*** (mg/mL)	LOQ (mg/mL)	Green tea**** (mg/cup)
GC	10.035 ± 0.021	0.1653	0.972	-	4852 ± 427	-36 ± 21	0.985	0.025	0.075	Below LOQ
EGC	15.890 ± 0.028	0.2074	1.056	18.4	9600 ± 321	-24 ± 14	0.997	0.011	0.032	33.65 +/- 0.74
Caffeine	18.648 ± 0.026	0.2115	1.017	7.9	59231 ± 371	-10 ± 14	0.999	0.0015	0.0046	18.21 ± 0.36
C	19.694 ± 0.022	0.2161	1.044	2.85	17606 ±101	-5 ± 4	0.999	0.0019	0.0057	Below LOQ
EC	26.926 ± 0.019	0.2144	1.041	19.8	15871 ± 103	-3 4	0.999	0.0021	0.0065	9.09 ± 0.15
EGCG	27.922 ± 0.020	0.2188	1.053	2.7	30122 ± 117	-13 ± 4	0.999	0.0012	0.0039	36.96 ± 0.61
GCG	30.810 ± 0.022	0.2177	1.049	7.8	35812 ± 129	-12 ± 5	0.999	0.0012	0.0036	Close to LOD
ECG	41.214 ± 0.017	0.2389	1.055	26.9	43028 ±229	-12 ± 9	0.999	0.0018	0.0053	8.10 ± 0.26
CG	42.129 ± 0.015	0.2371	1.037	2.27	45621 ± 260	-11 ± 10	0.999	0.0019	0.0057	Below LOD

*Evaluated for 0.04 mg/mL standard, **Calibration range 0.08-0.0025 mg/mL; Calibration equation: Peak area = slope X concentration (mg/mL) + intercept; ***The limit of detection (LOD = 3 σ_{y0}) and limit of quantification (LOQ = 10 σ_{y0}) were determined via the intercept's standard deviation of the calibration curve, σ_{y0}, with $\sigma_{y0} = \sqrt{n}\, SE_{yo}$(n=6). ****Catechin and caffeine content of Lipton tea sample purchased in Fall 2019 (n = 3) in mg/cup (235 mL))

Supplemental Table S2: HPLC parameters for the PFP Poroshell 120 column (4.6 x 250 mm, 4 µm particle size)

Compound	Retention time (min)	Peak width*	Peak symmetry	Resolution	Slope**	Intercept	R-square value	LOD*** (mg/mL)	LOQ (mg/mL)	Green tea **** (mg/cup)
GC	14.342 ± 0.048	0.1993	0.973	-	13224 ± 723	-12 ± 18	0.985	0.012	0.038	Below LOQ
Caffeine	18.195 ± 0.043	0.197	1.015	16.4	58627 ± 1802	-23 ± 44	0.995	0.0077	0.023	18.9 ± 1.01
EGC	20.072 ± 0.049	0.1911	0.928	11.5	3192 ± 91	-2 ± 2	0.996	0.0059	0.018	37.50 +/- 2.70
C	23.995 ± 0.043	0.2098	0.946	5.6	16022 ± 454	-9 ± 11	0.996	0.0059	0.018	Below LOQ
EC	29.001 ± 0.051	0.2059	0.943	11.5	14862 ± 427	-10 ± 10	0.996	0.0059	0.018	9.1 ± 0.58
EGCG	35.513 ± 0.065	0.2354	0.701	14.2	22342 ± 589	-17 ± 13	0.998	0.0077	0.023	37.4 ± 2.69
GCG	38.266 ± 0.153	0.2656	0.576	17.3	24239 ± 1279	-45 ± 33	0.990	0.010	0.032	Below LOD
ECG	46.373 ± 0.068	0.2544	0.806	6.3	35977 ± 1245	-44 ± 33	0.996	0.0070	0.021	8.5 ± 0.51
CG	48.640 ± 0.069	0.2803	0.603	18.5	17394 ± 974	-103 ± 30	0.994	0.0092	0.028	Not detected

*Evaluated for 0.04 mg/mL standard, **Calibration range 0.08-0.0025 mg/mL; Calibration equation: Peak area = slope X concentration (mg/mL) + intercept; ***The limit of detection (LOD = 3 σ_{y0}) and limit of quantification (LOQ = 10 σ_{y0}) were determined via the intercept's standard deviation of the calibration curve, σ_{y0}, with $\sigma_{y0} = \sqrt{n}\, SE_{yo}$(n=6). ****Catechin and caffeine content of Lipton tea sample purchased in Fall 2019 (n = 3) in mg/cup (235 mL)

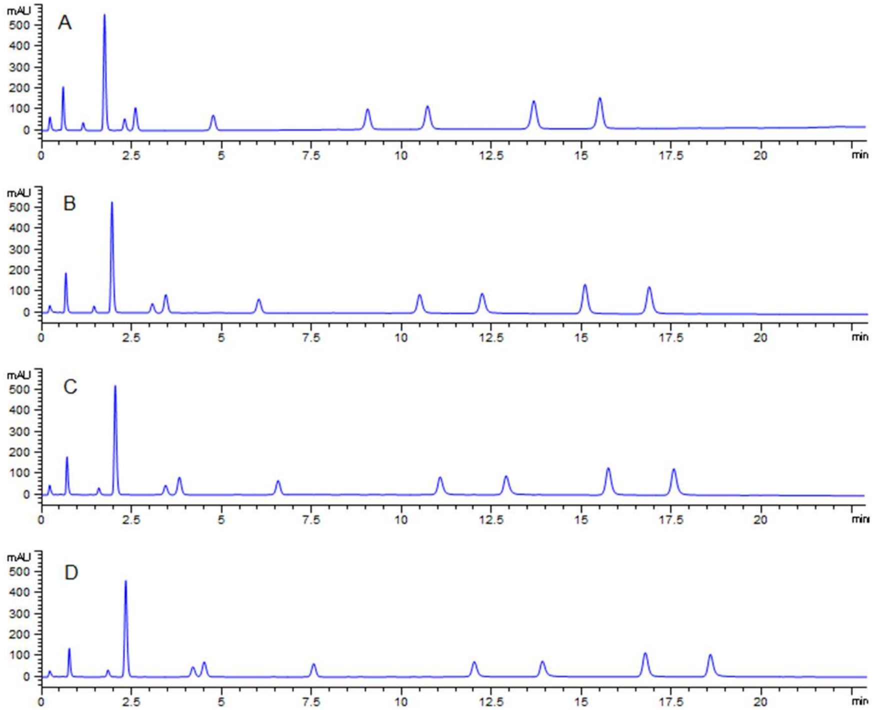

Figure S2: HPLC chromatograms of a green tea standard mixture recorded with a shorter InfinityLab Poroshell PFP column (2.1 x 50 mm, 1.9 µm particle size) with decreasing concentrations of formic acid in the mobile phase: 2 % (A), 1 % (B), 0.5 % (C), and 0.1 % (D). The peaks elute in the following order after the injection peak: gallic acid, GC, caffeine, EGC, C, EC, EGCG, GCG, ECG, CG.
